# Role of oxidative stress versus lipids in monocrotaline‐induced pulmonary hypertension and right heart failure

**DOI:** 10.14814/phy2.15090

**Published:** 2021-11-23

**Authors:** Firoozeh Farahmand, Akshi Malik, Anita Sharma, Ashim K. Bagchi, Pawan K. Singal

**Affiliations:** ^1^ Atlanta Georgia USA; ^2^ Institute of Cardiovascular Sciences St. Boniface Hospital Albrechtsen Research Centre Department of Physiology and Pathophysiology Rady Faculty of Health Sciences University of Manitoba Winnipeg Canada; ^3^ Research and Graduate Studies Thompson Rivers University Kamloops Canada

**Keywords:** antioxidants, oxidative stress, pulmonary hypertension, right heart failure

## Abstract

Pulmonary hypertension (PH) is a global health issue with a prevalence of 10% in ages >65 years. Right heart failure (RHF) is the main cause of death in PH. We have previously shown that monocrotaline (MCT)‐induced PH and RHF are due to an increase in oxidative stress. In this study, probucol (PROB), a strong antioxidant with a lipid‐lowering property, versus lovastatin (LOV), a strong lipid‐lowering drug with some antioxidant effects, were evaluated for their effects on the MCT‐induced RHF. Rats were treated (I.P.) with PROB (10 mg/kg ×12) or LOV (4 mg/kg ×12), daily 6 days before and 6 days after a single MCT injection (60 mg/kg). Serial echocardiography was performed and at 4‐week post‐MCT, lung wet‐to‐dry weight, hemodynamics, RV glutathione peroxidase (GSHPx), superoxide dismutase (SOD), catalase, lipid peroxidation, and myocardial as well as plasma lipids were examined. MCT increased RV systolic and diastolic pressures, wall thickness, RV end diastolic diameter, mortality, and decreased ejection fraction as well as pulmonary artery acceleration time. These changes were mitigated by PROB while LOV had no effect. Furthermore, PROB prevented lipid peroxidation, lowered lipids, and increased GSHPx and SOD in RV myocardium. LOV did decrease the lipids but had no effect on antioxidants and lipid peroxidation. A reduction in oxidative stress and not the lipid‐lowering effect of PROB may explain the prevention of MCT‐induced PH, RHF, and mortality. Thus targeting of oxidative stress as an adjuvant therapy is suggested.

## Graphical abstract

Pulmonary Hypertension (PH) is a progressive cardiovascular disease particularly in 65 years of age or older patients. Present study on monocrotaline‐induced PH model suggests that controlling oxidative stress reduces PH and improves RV dysfunction, RH failure, as well as mortality. Our laboratory data raise a strong possibility for a drug design which has antioxidant capability for addressing of the PH and RV dysfunction.

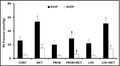

## 1 INTRODUCTION

Pulmonary hypertension (PH) is a progressive, devastating, and complex cardiovascular disease with an estimated global prevalence of 1%, increasing to 10% in 65 years and older people (Hirani et al., 2020). It is characterized by endothelial dysfunction, vascular remodeling, right ventricular (RV) hypertrophy, with subsequent right heart failure (RHF) resulting in premature death (Humbert et al., 1995; Martin et al., 2006). In PH, the 5‐year survival for patients with stable or improving RV function is >90%; whereas survival for patients with decreasing RV function is <30%, with RHF being the most common cause of death. Epidemiological studies have demonstrated that survival is significantly associated with changes in RV ejection fraction (RVEF), and much less with changes in peripheral vascular resistance (PVR) or pressures (Zelt et al., 2019). RV dysfunction is a complex clinical syndrome and is the strongest predictor of mortality in PH patients (Hoeper et al., 2016; Prisco et al., 2020). Despite the progress in our understanding of RV function and failure, significant gaps remain in the description of cellular mechanisms responsible and the development of any evidence‐based therapy (Konstam et al., 2018; Lahm et al., 2018).

In the last three decades there has been >40% improvement in the mortality of patients with left ventricular systolic dysfunction but many targeted therapies for left heart failure (LHF) do not improve RHF and there are no clinically approved therapies that either directly or selectively improve RV function (Vasan et al., 2018). Therefore, it is of crucial importance to investigate cellular mechanism involved in the pathophysiology of transition from adaptive to maladaptive stage of RV hypertrophy to RHF, that is, the point of no return. As we learn more about the transition from adaptive remodeling to maladaptive RHF, more successful strategies to manage RHF will be developed. Our previous studies and others have suggested oxidative stress as the final common pathway in this transition (Farahmand et al., 2004; Woo et al., 2017). In a previous study, we showed that at 2‐week post‐monocrotaline (MCT)‐induced PH and RV hypertrophy without any RHF were associated with increased activities of the antioxidant enzymes: superoxide dismutase (SOD); glutathione peroxidase (GSHPx); and catalase in the RV myocardium (Farahmand et al., 2004). Interestingly, at this stage, there was no evidence of an increase in oxidative stress in the RV myocardium. Thus, the increase in antioxidant reserve appears to be an adaptive mechanism to counteract stress. However, at 6‐week post‐MCT, RHF and mortality occurred with a concomitant significant reduction in antioxidant activities. Thus, an imbalance between oxidant stress and antioxidant reserve may have an important role in the pathogenesis of PH‐induced RV remodeling, failure, and death.

Probucol (PROB), a potent antioxidant with lipid‐lowering effects has been used in various cardiovascular diseases (Li & Singal, 2000; Singal & Iliskovic, 1998; Singla et al., 2007; Siveski‐Iliskovic et al., 1994; Yamashita & Matsuzawa, 2009). An improvement in inflammatory and antioxidative response by PROB with a reduction in atherosclerosis has been reported (Bräsen et al., 2002; Zhang et al., 2018). In another study, PROB was shown to increase the expression of heme oxygenase‐1, a redox‐sensitive enzyme (Wu et al., 2006). Clinical trials and meta‐analysis demonstrated that PROB is effective in reducing the risk of restenosis and incidence of major adverse cardiac events after percutaneous transluminal intervention (Liu et al., 2015). Furthermore, PROB has also been shown to promote the growth of endothelial cells and arterial relaxation and functional re‐endothelization following aortic balloon injury in rabbits (Lau et al., 2003). PROB protects endothelial progenitor cells against oxidized low‐density lipoprotein via suppression of reactive oxygen species (Zhang et al., 2016). PROB is, however, a relatively weak lipid‐lowering drug when compared to statins (Yamashita & Matsuzawa, 2009). Lovastatin (LOV), inhibitor of HMG‐CoA reductase, is a well‐established lipid‐lowering drug with some antioxidant properties (Kumar et al., 2011) but lower in vivo antioxidant effect (Hussein et al., 1997).

Thus, in the present study, we used PROB and LOV to investigate whether antioxidant property of PROB or the lipid‐lowering effect of LOV, or both were important in the anticipated beneficial effects of these drugs in mitigating MCT‐induced PH. Specifically, the study focused on: (i) Whether treatment with PROB or LOV will prevent RV hypertrophy and RV pressure overload and (ii) Would these treatments modify endogenous antioxidants and oxidative stress. In addition to the hemodynamic data, the activity of myocardial antioxidants enzymes, glutathione peroxidase (GSHPx); superoxide dismutase (SOD); and catalase (CAT), as well as oxidative stress, plasma, and myocardial lipid levels were also examined.

## 2 MATERIALS AND METHODS

All animal experimental protocols were approved by the University of Manitoba Animal Care Committee following the guidelines established by the Canadian Council on Animal Care and conform to the NIH Animal care Guide (NIH Publication No. 85‐23, revised 1996).

### 2.1 Animal groups

Male Sprague Dawley rats (*n* = 60; BW, 220 ± 10 g) were divided equally into six groups: (1) control (CONT); (2) monocrotaline (MCT) alone; (3) probucol (PROB) alone; (4) MCT+ +PROB; (5) lovastatin (LOV) alone; and (6) MCT + LOV. MCT buffered at pH 7.0 was administered as a single 60 mg/kg I.P. injection. PROB was administered daily (10 mg/kg, I.P.) 6 days prior and 6 days post‐MCT injection for a cumulative dose of 120 mg/kg in 12 equal injections. Same schedule and route of injection were used for the LOV treatment for a cumulative dose of 48 mg/kg (4 mg/kg daily ×12). The drug dosages were based on our previous studies (Farahmand et al., 2004; Siveski‐Iliskovic et al., 1994). Animals were observed daily for their general body conditions, any clinical signs of respiratory distress, and weighed. Following studies were performed at 4‐week post‐MCT injection.

### 2.2 Lung wet‐to‐dry weight ratios

Pieces of tissue from the lungs were removed and weighed to obtain the wet/dry weight ratio. For recording the dry weight, pre‐weighed tissue was chopped into smaller pieces and was placed in an oven at 65°C until a constant weight was reached.

### 2.3 Echocardiographic measurements

Echocardiographic studies were performed using a 5500 ultrasound system with 2.5, 3.5, and 10 MHZ transducers in accordance with the standards of the American Society of Echocardiography at 4‐week post‐MCT injection (Hardziyenka et al., 2006). All images were recorded and subsequently analyzed using software packages. Doppler velocity curves were recorded at sweep velocity of 100 cm/s and pulmonary artery acceleration time (PAAT), the time from the beginning to the peak of the velocity envelop during pulsed Doppler interrogation of the pulmonary valve. Right ventricle end diastolic diameter (RVEDD), right ventricle wall thickness (RVWT), and ejection fraction (EF) were measured as previously described (Hardziyenka et al., 2006). Each parameter was averaged over three cardiac cycles.

### 2.4 Hemodynamic measurements

Animals were anesthetized with ketamine and xylazine, 60 and 10 mg/kg, respectively, I.P. For recording of right ventricular (RV) pressure, a miniature pressure transducer (Millar Microtip, Model PR 249) was inserted into the right jugular vein and then advanced to the RV. Using specific software (Windows Biopac Systems Inc.), right ventricular peak systolic pressure (RVSP) and right ventricular peak diastolic pressure (RVDP) were recorded. After these assessments, animals were sacrificed and heart, lung, and plasma were collected for further studies. Hearts were immediately washed in 0.033 M Na_2_HPO_4_ and 0.9% KCl buffer for further analysis.

### 2.5 Antioxidant enzymes and oxidative stress

For GSHPx, RV was homogenized in 10 volumes of 75 mmol/L phosphate buffer, pH 7.0. Homogenate was centrifuged at 18,000 g for 45 min and the supernatant was aspirated and assayed according to the procedure described before (Rakotoniaina et al., 2006). GSHPx activity was expressed as nanomoles of NADPH converted to NADP per min per mg protein, with a molar extinction coefficient for NADPH at 340 nm of 6.22 × 10^6^ (Paglia & Valentine, 1967). For superoxide dismutase (SOD), the RV homogenate was centrifuged (20,000*g* for 20 min) and the supernatant was assayed for SOD activity by following the inhibition of pyrogallol autoxidation (Marklund, 1985). Catalase (30 μM stock solution) was added to Tris–HCl buffer containing 25 μl of pyrogallol and 10 μl of catalase. The final 3 ml was made up with the same buffer. Changes in absorbance at 420 nm were recorded at 1‐min intervals for 5 min. SOD activity was determined from a standard curve of percent inhibition of pyrogallol autoxidation with a known SOD activity. Data were expressed as SOD units per mg protein as compared with the standard. For catalase, the RV was homogenized in 9 volumes of 0.05 M potassium phosphate buffer (pH 7.4) and centrifuged at 40,000*g* for 30 min. Supernatant (50 μl) was added to the cuvette containing 2.95 ml of 19 mM H_2_O_2_ solution prepared in potassium phosphate buffer. The color was read at 240 nm on a Spectronic 601 every min for 5 min. Commercially available catalase was used as a standard. Specific activity of the enzyme was expressed as units per mg protein (Clairborne & Greenwald, 1985). For the analysis of oxidative stress, lipid peroxidation was measured in the myocardium by determining thiobarbituric acid‐reactive (TBA) substances as described before (Singal & Pierce, 1986). The developed color in the supernatant was read at 532 nm on a spectrophotometer. Commercially available malondialdehyde was used as the standard.

### 2.6 Lipid profile

Plasma lipids (total cholesterol, high‐density (HDL), low‐density (LDL) lipoproteins, and triglycerides) were determined enzymatically using kits obtained from Sigma diagnostics (352, 352–3, and 336, Sigma Chemical Co.) and expressed as mg per deciliter of plasma. For the myocardial studies, the RV was homogenized in 10 ml of 0.05 mol/L potassium phosphate buffer (pH 7.4) and centrifuged at 40,000*g* for 30 min. Supernatants were assayed using the kits and expressed as mg per gm of tissue.

### 2.7 Proteins and statistical analysis

Proteins were determined (Lowry et al., 1951) and data are expressed as the mean ± SEM. For the statistical analysis, group means were compared by one‐way analysis of variance (ANOVA), and data from the groups were compared by Bonferroni's post hoc test. Statistical significance was acceptable at the level of *p* ≤ 0.05.

## 3 RESULTS

### 3.1 General observations

All animals were observed for 4 weeks after the MCT injection. Animals in the CONT, PROB, and LOV groups showed a significant increase in the weight with an average gain of 175 ± 25 g over a period of 4 weeks. However, animals in the MCT group did not gain weight over this period. Both the PROB + MCT and LOV + MCT groups also did not show any weight gain. One week after treatment, MCT‐treated rats showed signs of hypoactivity, fatigue, and intercoastal muscle retraction. The most noticeable characteristic of rats in the MCT group was the development of respiratory distress which became apparent between 2nd and 3rd weeks after MCT treatment. After 3 weeks, in the MCT group, there was engorgement of the jugular vein associated with peripheral cyanosis. These signs persisted and got aggravated in the 4th‐week post‐MCT treatment period resulting in 30% mortality. PROB treatment completely prevented these MCT‐induced changes, whereas LOV had no beneficial effect.

### 3.2 Lung wet‐to‐dry weight ratio

There was an increase in the wet‐to‐dry weight ratio for lungs in the MCT group (CONT, 3.0 ± 0.04; MCT, 7.0 ± 0.07). In PROB‐treated MCT group, this change was significantly less. However, LOV failed to prevent this MCT‐induced increase in lung wet‐to‐dry weight ratio. There was no significant change in this ratio in any of the other groups (data not shown).

### 3.3 Right ventricle function

**FIGURE 1:**
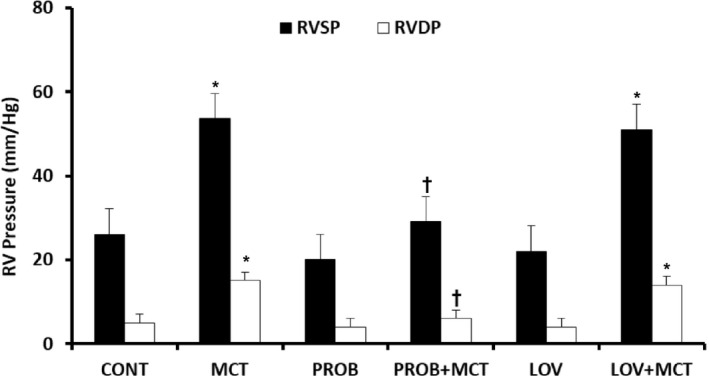
Effect of probucol (PROB) and lovastatin (LOV) on monocrotaline (MCT)‐induced changes in right ventricle systolic pressure (RVSP) and right ventricular diastolic pressure (RVDP) in rats at 4‐week post‐MCT treatment. Data are mean ± SEM of 9–12 animals. *Significantly different (*p* < 0.05) from the respective controls (CONT); ^†^Significantly different (*p* < 0.05) from the respective values in the MCT group

Animals in all the groups were also assessed for the right ventricle function (systolic and diastolic pressures) at 4‐week post‐MCT treatment. There was a significant increase in RVSP and RVDP in MCT group as compared to respective CONT groups (Figure 1). Treatment with PROB prevented MCT‐induced increase in both RVSP and RVDP in PROB + MCT group. Such a protection was not seen due to LOV in the LOV + MCT group. PROB or LOV by itself had no effect on the right heart function.

Furthermore, there was a significant increase in RVWT and RVEDD as well as a decrease in the EF at 4‐week post‐MCT in the MCT group (Table 1) as compared to CONT group. PROB treatment was able to attenuate these changes in PROB + MCT group. LOV had no effect on these MCT‐induced changes in the RV in the LOV + MCT group (Table 1).

**TABLE 1:** Effect of probucol and lovastatin on monocrotaline‐induced changes in RVEDD, RVWT, and EF at 4‐week post‐MCT treatment in rats

Group	RVEDD (mm)	RVWT (mm)	EF (%)
CONT	4.1 ± 0.1	0.65 ± 0.01	48 ± 08
MCT	6.2 ± 0.1*	0.90 ± 0.01*	34 ± 14
PROB	4.3 ± 0.1	0.63 ± 0.01	46 ± 12
PROB + MCT	4.5 ± 0.1^†^	0.67 ± 2.00^†^	46 ± 11
LOV	4.3 ± 0.1	0.62 ± 0.01	46 ± 12
LOV + MCT	6.2 ± 0.1*	0.88 ± 0.01*	36 ± 10

Note

Data are mean ± SEM from 5 to 6 rats.

Abbreviations: EF, ejection fraction; LOV, lovastatin; MCT, monocrotaline; PROB, probucol; RVEDD, right ventricle end diastolic diameter; RVWT, right ventricle wall thickness.

* Significantly different (*p* < 0.05) from the control (CONT) and the PROB + MCT groups.

^†^ Significantly different (*p* < 0.05) from the MCT and the LOV + MCT groups.

**FIGURE 2:**
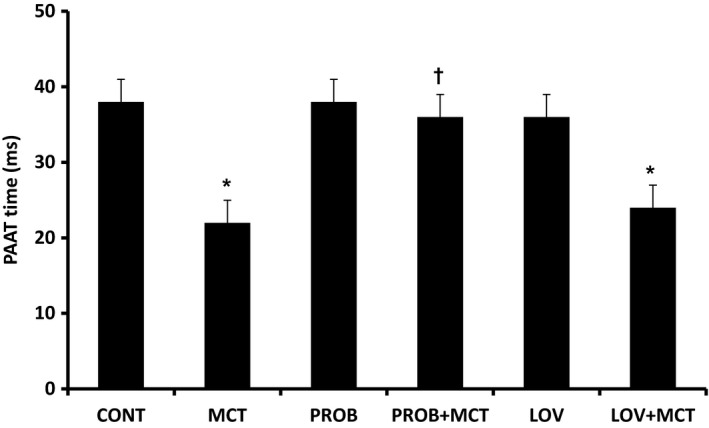
Effect of probucol (PROB) and lovastatin (LOV) on monocrotaline (MCT)‐induced changes in pulmonary artery acceleration time (PAAT) in rats at 4‐week post‐MCT treatment. Acceleration time was measured from the time of onset of systolic flow to peak pulmonary outflow. Data are mean ± SEM of 9–12 animals. ^*^Significantly different (*p* < 0.05) from the control (CONT) group; ^†^Significantly different (*p* < 0.05) from the MCT group

Pulmonary artery acceleration time (PAAT) was recorded in all the groups (Figure 2). MCT group showed a significant decrease in PAAT indicative of increase in pulmonary artery pressure as compared to CONT group at 4‐week post‐MCT treatment. In PROB + MCT group, PAAT was comparable to that of CONT group, however LOV was unable to prevent this MCT‐induced change in PAAT in the LOV + MCT group. PROB and LOV by itself had no effect on PAAT.

### 3.4 Oxidative stress and antioxidant enzymes

**FIGURE 3:**
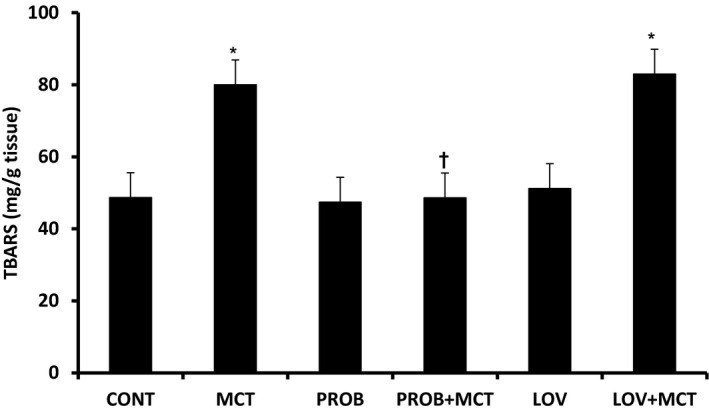
Effect of probucol (PROB) and lovastatin (LOV) on monocrotaline (MCT)‐induced changes in lipid peroxidation assessed by thiobarbituric acid‐reactive substances (TBARS) in rats at 4‐week post‐MCT treatment. Data are mean ± SEM of 5–6 animals. ^*^Significantly different (*p* < 0.05) from the control (CONT) group; ^†^Significantly different (*p* < 0.05) from the MCT and LOV + MCT groups

To assess oxidative stress in the RV, lipid peroxidation, and endogenous antioxidant enzymes, GSHPx, SOD, and CAT, were analyzed in all the groups at 4‐week post‐MCT treatment (Table 2; Figure 3). Lipid peroxidation in the RV was significantly increased in MCT group. PROB treatment brought the lipid peroxidation to control levels in the PROB + MCT group, whereas LOV treatment had no effect on the MCT‐induced increase in the lipid peroxidation (Figure 3). PROB and LOV by itself had no effect on lipid peroxidation.

**TABLE 2:** Effect of probucol and lovastatin on monocrotaline‐induced changes in right ventricle antioxidant enzyme activities at 4‐week post‐MCT treatment in rats

Group	GSHPx (nmol/mg protein)	SOD (U/mg protein)	CAT (U/mg protein)
CONT	44.7 ± 4.6	36.4 ± 2.0	29.3 ± 2.8
MCT	23.6 ± 3.4*	34.2 ± 3.8	31.4 ± 3.1
PROB	50.4 ± 2.4	52.6 ± 5.4^†^	35.4 ± 2.7
PROB + MCT	62.6 ± 4.2^†^	59.3 ± 4.0^†^	27.3 ± 3.2
LOV	41.2 ± 3.4	37.6 ± 2.6	31.4 ± 3.7
LOV + MCT	24.4 ± 2.4*	42.3 ± 3.7	33.6 ± 4.2

Note

Data are mean ± SEM of 5–6 animals.

Abbreviations: CAT, catalase; GSHPx, glutathione peroxidase; SOD, superoxide dismutase.

* Significantly different (*p* < 0.05) from the CONT, PROB, PROB + MCT, and LOV groups.

^†^ Significantly different (*p* < 0.05) from the CONT, MCT, LOV, and LOV + MCT groups. All other legends are similar to that in Table 1.

MCT treatment significantly reduced the levels of GSHPx and PROB treatment significantly improved the levels of GSHPx in PROB + MCT group and the level was even higher than the CONT group. However, LOV treatment had no effect on the GSHPx level in the LOV + MCT group as compared to MCT group (Table 2). PROB and LOV by itself had no effect on this activity. MCT and LOV treatments did not affect SOD levels, whereas PROB significantly increased the activities of SOD, in the PROB as well as in the PROB + MCT group. The activity of CAT was not affected by MCT or PROB or LOV treatment (Table 2).

### 3.5 Lipids in plasma and right ventricle

Total cholesterol, HDL, and LDL as well as triglycerides were measured in the plasma (Table 3). MCT caused a significant increase in plasma total cholesterol, HDL, LDL, and triglycerides (Table 3). PROB treatment significantly reduced these MCT‐induced increase in total cholesterol, HDL, LDL, and triglycerides in the PROB + MCT group, but the values were not back to the control levels. However, HDL levels were reduced below the control levels in PROB and PROB + MCT groups (Table 3). In the PROB alone group, there was a tendency toward a decrease in all lipids. However, the decrease was significant only in the HDL, LDL, and triglycerides levels as compared with CONT group. LOV treatment significantly reduced total cholesterol, LDL as well as triglycerides changes due to MCT, but the values were not back to the normal levels. LOV alone increased HDL level which was significantly different from CONT group. However, it did not affect other plasma lipids (Table 3).

**TABLE 3:** Effects of probucol and lovastatin on monocrotaline‐induced changes in plasma lipids at 4‐week post‐MCT treatment in rats

Group	Total cholesterol (mg/dl)	HDL (mg/dl)	LDL (mg/dl)	Triglycerides (mg/dl)
CONT	80 ± 12.1	30 ± 6.0	36 ± 5.2	200 ± 17.7
MCT	400 ± 25.6*	51 ± 7.9*	150 ± 5.6*	702 ± 9.0*
PROB	71 ± 5.2	14 ± 0.8*	23 ± 3.4*	150 ± 12.8*
PROB + MCT	123 ± 15.6* ^,^ ^†^	18 ± 3.6* ^,^ ^†^	80 ± 7.6* ^,^ ^†^	301 ± 26.5* ^,^ ^†^
LOV	86 ± 6.3	50 ± 4.8*	41 ± 4.3	230 ± 49.6
LOV + MCT	187 ± 6.3* ^,^ ^†^	46 ± 6.34*	46 ± 3.4^†^	570 ± 31.3* ^,^ ^†^

Note

Data are mean ± SEM from 5 to 6 experiments. All other legends are similar to that in Table 1.

Abbreviations: HDL, high‐density lipoproteins; LDL, low‐density lipoproteins.

* Significantly different (*p* < 0.05) from the CONT group.

^†^ Significantly different (*p* < 0.05) from the MCT group.

Total cholesterol and triglycerides were also measured in the RV (Table 4) in all the groups at 4‐week post‐MCT treatment. MCT significantly increased total cardiac cholesterol and triglycerides levels (Table 4). Both PROB and LOV treatments normalized these MCT‐induced changes in cardiac lipids. PROB and LOV alone significantly decreased total cholesterol level compared with CONT but did not affect triglyceride level (Table 4).

**TABLE 4:** Effects of probucol and lovastatin treatment on monocrotaline‐induced changes in right ventricle at 4‐week post‐MCT treatment in rats

Group	Total cholesterol (mg/g)	Triglycerides (mg/g)
CONT	17.87 ± 2.30	30.56 ± 3.04
MCT	28.32 ± 2.45*	51.34 ± 4.70*
PROB	9.23 ± 0.78*	31.67 ± 3.17
PROB + MCT	14.32 ± 1.20^†^	31.30 ± 3.24^†^
LOV	8.43 ± 0.06*	31.43 ± 3.26
LOV + MCT	12.53 ± 0.04^†^	27.45 ± 3.02^†^

Note

Data are mean ± SEM of 5–6 animals. All other legends are similar to that in Table 1.

* Significantly different (*p* < 0.05) from the CONT group.

^†^ Significantly different (*p* < 0.05) from the MCT group.

## 4 DISCUSSION

PH remains a progressive and fatal disease despite the availability of pharmaceutical therapies. Current drugs improve the quality of life and hemodynamic parameters but have very limited beneficial effects on survival and progression of the disease. RHF is the single most predictor of mortality in PH. Although RHF management remains supportive, symptom‐based and specific therapies promoting stabilization and recovery of RV function are lacking.

The present study shows that MCT‐induced PH is associated with not only RV hypertrophy but also increased RVDP and RV dilatation indicative of RV dysfunction. These changes were accompanied by 30% mortality. There was an increase in oxidative stress as suggested by increased lipid peroxidation as well as reduced antioxidant reserve in the right ventricular myocardium. Oxidative stress is an important mechanism of vascular and cardiac injury. Experimental as well as clinical studies have indicated that oxidative stress is implicated in the pathogenesis of cardiac dysfunction, ischemia‐reperfusion injury, hypertrophy, cell death, and HF. Hydroxyl radicals originating from superoxide and H_2_O_2_ in the failing hearts can damage the myocardium (Singal & Kirshenbaum, 1990; Slezak et al., 2021; Takimoto & Kass, 2007). It has been shown that oxidative stress induces apoptosis of cardiomyocytes with adverse effect on ventricular function leading to heart failure (Akolkar et al., 2017; Zheng et al., 2020).

Although there are numerous studies highlighting the structure and/or function changes in the RV secondary to PH, the molecular pathways that regulate these changes in the RV are not well studied (Lahm et al., 2018). We have already shown that MCT generates free radicals and increases oxidative stress in the RV (Farahmand et al., 2004). Oxidative stress mediates morphological changes in pulmonary vasculature and plays a key role in pulmonary vascular remodeling, RV hypertrophy, and RHF as well as in overall heart failure (Aziz et al., 1997; Farahmand et al., 2004; Ludke et al., 2010; Zimmer et al., 2021:^891^:^173699^.). In clinical studies also, the lungs of patients with severe pulmonary hypertension were under chronic oxidative stress and there was a decrease in the activity of MnSOD (Bowers et al., 2004). Furthermore, in other experimental models of PH, oxidative stress markers have been associated with the RV remodeling. In SOD3 knockout mouse model with silica‐induced PH, authors showed that a decrease in SOD3 resulted in an imbalance between oxidants and antioxidants as well as led to the vascular remodeling, PH, and RV pressure overload (Zelko et al., 2018). The isoform SOD‐2 reduces superoxide anion to hydrogen peroxide, which is less likely to damage the RV vasculature (Maron & Abman, 2017). Recently, in an experimental hypoxia model of PH, it was shown that insufficient extracellular SOD3 disrupts redox balance and contributes to the pathogenesis of PH (Tseng et al., 2020).

In the present study, PROB an antioxidant and a lipid‐lowering agent, prevented MCT‐induced PH, RVH, and RVF. PROB also increased the activities of GSHPx and SOD as well as reduced oxidative stress in the right ventricle. It is known that GSHPx removes H_2_O_2_ formed by catalytic activity of SOD and also through the detoxification of lipid hydroperoxides (Shiomi et al., 2004; Singal et al., 1987). Another important aspect of this study is that the PROB treatment was given only up to 1‐week post‐MCT treatment, but the increased activities of GSHPx and SOD were sustained up to 4‐week post‐MCT treatment. Upregulation of endogenous SOD by PROB in the RV has been reported to be important as during the transition from RV hypertrophy to RV failure, the RV mitochondrial SOD‐2 was downregulated (Redout et al., 2007). Furthermore, it has recently been shown in patients that there is an independent link of serum EC‐SOD activity with abnormal LV geometry patterns with and without overt HF (Li et al., 2020). Furthermore, in diabetic hearts, elevating MnSOD provided extensive protection to mitochondria as well as overall protection (Shen et al., 2006). PROB has also been shown to prevent the increase in circulating malondialdehydes in rats with myocardial infarction (Sia et al., 2002). In the present study also, there was a significant increase in the malondialdehyde in the MCT group which was prevented by PROB. Thus, PROB is not only an antioxidant, but it also promotes endogenous antioxidants and improves antioxidant reserve as well as decreases oxidative stress (Singal et al., 1987).

Interestingly, MCT‐induced PH was associated with an increase in the myocardial plasma triglycerides, total cholesterol, HDL, and LDL levels. Both PROB and LOV mitigated these MCT‐induced changes in the lipids. However, unlike PROB, LOV had no effect on the MCT‐induced PH, RVH, RVF, and mortality. PROB also caused a significant reduction in the HDL levels, which may be of concern. However, this HDL lowering effect is a part of the trade off in the overall beneficial effect of probucol through the lowering of LDL. In fact, studies suggest the concept of HDL metabolism that shows PROB promotes cholesterol efflux and enhances reverse cholesterol transport by activation of cholesteryl ester transfer protein and scavenger receptor class B type I which may improve HDL function despite lowering HDL‐C (Yamashita & Matsuzawa, 2009). Here, it should be noted that LOV had no mitigating effect on MCT‐induced decrease in antioxidants as well as increase in oxidative stress. These data support our contention that lipid‐lowering property of PROB may not have a significant role in its beneficial effects. Similar to our study, others have also reported inability of some of the statins in the protection against MCT‐induced hypertension (Rakotoniaina et al., 2006). Meta‐analysis of 8 studies with 665 patients did not suggest any statistically significant effect of statin therapy in the improvement of pulmonary arterial pressure, right atrial pressure, cardiac index, and pulmonary vascular resistance. (Rysz‐Górzynska et al., 2016).

It appears that MCT‐induced PH is mediated by oxidative stress and PROB mitigates this effect by increasing the activities of GSHPx and SOD as well as lowering lipid peroxidation in the RV and likely in the pulmonary vasculature as well. PROB not only promotes endogenous antioxidants but also is a stronger antioxidant by virtue of the two phenolic groups in its molecular structure which help in the quenching of the free radicals (Singal et al., 1987). At least in the present study, LOV did not exert any antioxidant effect and thus was unable to mitigate MCT‐induced oxidative stress, PH, and RV failure. It is suggested that increase in oxidative stress plays a causal role in MCT‐induced RHF while an increase in both myocardial and plasma lipids may be a co‐event. Our results suggest that increasing the endogenous antioxidant reserve in patients with PH may prevent or halt the progression of RV failure. Thus, PROB as an adjunct therapy in PH may be potentially beneficial and needs a further follow‐up.

## CONFLICT OF INTEREST

There is no conflict of interest and no relationship with industry to disclose.

## AUTHOR CONTRIBUTIONS

F.F. and P.K.S. contributed to experimental design. F.F. performed the experiments and did data analysis. P.K.S. provided resources and materials. F.F., P.K.S., A.M., A.S., and A.K.B. contributed to writing, editing, and final submission of the manuscript.
